# Expression of osteopontin coregulators in primary colorectal cancer and associated liver metastases

**DOI:** 10.1038/bjc.2011.33

**Published:** 2011-02-22

**Authors:** D J Mole, C O'Neill, P Hamilton, B Olabi, V Robinson, L Williams, T Diamond, M El-Tanani, F C Campbell

**Affiliations:** 1 Clinical and Surgical Sciences (Surgery), The University of Edinburgh, Edinburgh EH8 9YL, Scotland, UK; 2 MRC Centre for Inflammation Research, Queen's Medical Research Institute, The University of Edinburgh, Edinburgh EH8 9YL, Scotland, UK; 3 Department of Pathology, Royal Group of Hospitals NHS Trust, Royal Group of Hospitals Trust, Belfast, BT12 6BJ, UK; 4 Public Health Sciences, Teviot Place, The University of Edinburgh, Edinburgh EH8 9YL, Scotland, UK; 5 Mater Hospital, Crumlin Road, Belfast, Northern Ireland; 6 Centre for Cancer Research and Cell Biology, Queen's University of Belfast, Lisburn Road, Belfast, BT9 7BL, UK

**Keywords:** osteopontin, colorectal cancer, metastasis, hepatectomy

## Abstract

**Background:**

A transcription regulatory complex (TRC) that includes Ets1, Ets2, PEA3 and *β*-catenin/T-cell factors regulates osteopontin (OPN) that is implicated in colorectal cancer (CRC) dissemination. The consistency of OPN transcriptional control between primary CRC and metastases is unclear. This study investigates expression and prognostic significance of the OPN–TRC in primary human CRC and associated colorectal liver metastases (CRLM).

**Methods:**

Osteopontin–TRC factors were assayed by digital microscopy in 38 primary CRCs and matched CRLM specimens and assessed against clinical prognosis.

**Results:**

In primary CRC, OPN expression intensity correlated with that of its co-activators, PEA3 (*r*=0.600; *P*<0.01), Ets1 (*r*=0.552; *P*<0.01), Ets2 (*r*=0.521; *P*<0.01) and had prognostic significance. Osteopontin intensity in primary CRC inversely correlated with the interval between diagnosis and resection of CRLM. Overall OPN intensity was lower in CRLM than primary CRC and correlations with co-activators were weaker, for example, Ets1 (*P*=0.047), PEA3 (*P*=0.022) or nonsignificant (Ets2). The ratio of OPN expression in CRLM *vs* primary CRC had prognostic significance.

**Conclusion:**

This study supports transcriptional control of OPN by known coregulators in both primary and secondary CRC. Weaker associations in CRLM suggest involvement of other unknown factors possibly from the liver microenvironment or resulting from additional genetic or epigenetic changes that drive tumour metastatic capability in OPN transcriptional control.

Successful establishment of metastases from circulating tumour cells requires integration of cell-autonomous oncogenic signalling with external cues from the specific tissue microenvironment of target organs (Fidler, 2002). Osteopontin (OPN) promotes invasiveness of colorectal cancer (CRC) (Irby *et al*, 2004) and other solid tumours (El-Tanani *et al*, 2006) and is a lead marker of human CRC progression (Agrawal *et al*, 2002; Eschrich *et al*, 2005). Osteopontin is regulated by Ets transcription factors partly by specific binding to the OPN promoter region (Sato *et al*, 1998) and partly through crosstalk with Wnt and other signalling pathways (El-Tanani *et al*, 2004). Combinatorial effects of Ets-1, Ets-2, PEA3 and *β*-catenin/T-cell factors (*β*-catenin/Tcfs) enhance OPN expression (El-Tanani *et al*, 2004) although unbound Tcf-4 may act as a transcriptional OPN repressor (El-Tanani *et al*, 2006). In primary CRC, molecular consequences of the initiating adenomatous polyposis coli mutation (Morin *et al*, 1997) include hyperactivation of *β*-catenin/Tcf signalling (Polakis, 2000) that upregulates PEA3 (Liu *et al*, 2004). This deregulated signalling may contribute to OPN overexpression (El-Tanani *et al*, 2004).

In addition to the molecular consequences of initiating mutations, the gene expression profiles of metastatic cells may be influenced by their supporting microenvironment in target organs (Nakamura *et al*, 2007). The consistency of the OPN-regulatory cassette between primary human CRC and related liver metastases, however, remains unclear. This issue has practical importance because surgery for liver metastases may be combined with a range of other therapeutic modalities (Garden *et al*, 2006) that could be guided by molecular profiling. Here, we assess expression and prognostic relevance of OPN-regulatory networks by immunohistochemistry and digital microscopy, in paired human primary CRCs and liver metastases.

## Patients and methods

### Patients

The study included 38 consecutive patients who underwent liver resection for colorectal liver metastases (CRLM) at the Mater Infirmorum Hospital Belfast between 1994 and 2003, for whom sufficient archive tissue was available. Clinicopathological characteristics were recorded including age, sex, site of colonic primary tumour, TNM classification and Duke's stage, date of colonic surgery, date of liver resection, number and segmental location of liver metastases, size of the largest liver metastasis, type of liver resection (left *vs* right, extended *vs* standard, anatomical *vs* non-anatomical atypical), resection margin status (R0 or other), immediate post-operative course (blood loss, blood transfusion, remnant liver function, extrahepatic organ failure, in-hospital mortality), details of adjuvant chemotherapy and date of death. Patients classified as Duke's D presented with synchronous metastases. Those with classification Duke's A to C developed metachronous metastases.

### Ethical approval

This study was approved by the Northern Ireland Research Ethics Committee.

### Immunohistochemistry

Archived formalin-fixed paraffin-embedded tissue blocks from the primary colon resection specimen and the matching resected CRLM were sectioned (5 *μ*m), mounted on APS-coated slides and anonymised by a coding system that preserved the within-patient matched-pair design. Immunohistochemical staining was automated on a DAKO autostainer (DAKO, Cambridgeshire, UK) through standard dewaxing, blocking, staining and washing protocols. Antigen retrieval was performed by microwaving in citrate buffer at high power for 10 min. Consecutive sections were stained with antibodies (at 1:100 dilution) to the following proteins: OPN (MAb MBIII B10 mouse/hybridoma, DHSB, University of Iowa, USA); *β*-catenin (H-102, sc-7199, Santa Cruz Biotechnology, Santa Cruz, CA, USA); Tcf4 (H-125, sc-13027, Santa Cruz Biotechnology); Ets-1 (H-150, sc-22802, Santa Cruz Biotechnology); Ets-2 (H-140, sc-22803, Santa Cruz Biotechnology); and PEA3 (H-120, sc-22806, Santa Cruz Biotechnology) or negative control where no primary antibody was used. Secondary visualisation was performed for all sections (including the no primary antibody controls), using DAKO Envision Plus HRP Kits (K4007, DAKO) according to the manufacturer's instructions.

### Digital assessment of gene expression and data analysis

**Figure 1:**
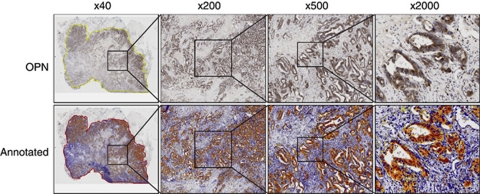
Digital expression analysis of OPN. An automated positive pixel count algorithm was applied to the digitally scanned annotated images. The image shown is a section of colorectal primary tumour stained for OPN visualised with DAB.

Slides were scanned digitally using an Aperio Scanscope CS2 (Aperio Technologies Inc., Vista, CA, USA) at × 40 objective magnification. Each slide was scanned in its entirety and stored as a jpeg format compressed tiff (svs) file (approximately 0.5 Gb per slide). Slide images were viewed using ImageScope (Aperio Technologies Inc.) and annotated digitally by accurately tracing the area of interest corresponding to tumour tissue. The annotated area was segmented using pixel density threshold analysis developed in-house and run within the ImageScope software (Figure 1). This used custom parameters detailed in Supplementary Table S1. The number and staining intensity of each pixel within the area of interest was calculated and exported for analysis via Microsoft Excel. Positivity was defined as the number of pixels exceeding the set threshold for staining intensity divided by the total number of pixels within the annotated area, expressed as a proportion. Intensity of staining was a continuous scale variable defined as the sum of staining intensities of all pixels within the annotated area. Subsequent statistical analysis and comparison with clinicopathological data was performed using SPSS v14.0 (SPSS Inc., Chicago, IL, USA). The relationship of immunohistochemical covariates (expressed as the ratio of staining intensity between colon and liver), clinical and pathological features to survival after hepatic resection was investigated by a Cox proportionate hazard model.

## Results

A total of 26 patients were male and 12 were female with median age of 62.1 years at colectomy (interquartile range, 12.8 years). Pre-operative liver synthetic function (albumin and prothrombin time) were within the normal reference range and all patients were seronegative for hepatitis B and C. Details of tumour primary site, Duke's classification and TNM stage are presented in Table 1. The median interval between colectomy for primary CRC and liver resection for metastatic disease was 1.51 years (95% CI: 0.84, 2.18 years). The site, number, size, resection margin of liver metastases are presented in Table 2. The median survival time from liver resection was 3.90 years (95% CI: 3.2, 4.6 years) and from colectomy was 4.95 years (95% CI: 4.05, 5.85 years). In all, 20% of patients remained free of detectable cancer at 5 years after liver resection.

**Figure 2:**
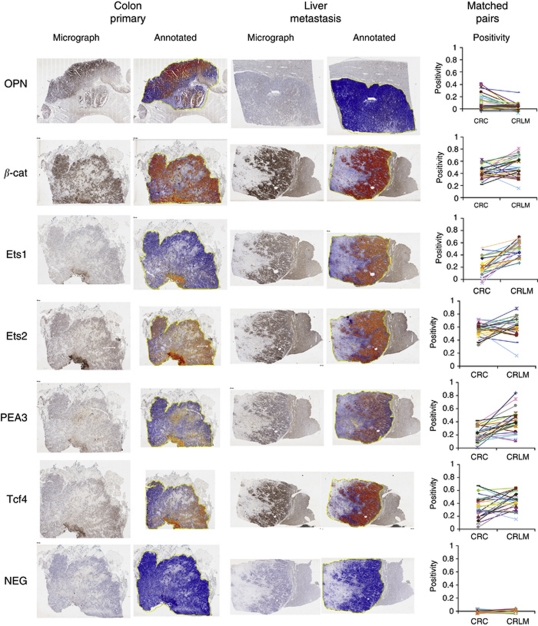
Osteopontin and coregulators in primary CRC and CRLM matched pairs. Consecutive sections of colorectal primary tumour and liver metastasis, illustrating the raw scanned image and the positive pixel count for each primary antibody. Line diagrams show the difference between primary tumour and matched liver metastasis for each patient.

**Figure 3:**
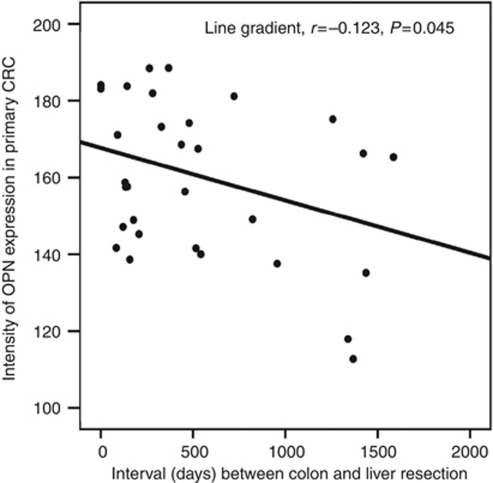
Osteopontin intensity in primary CRC *vs* interval to liver resection. Osteopontin expression in primary CRC inversely correlates with interval between colectomy and liver resection for metastases (*P*=0.045; Pearson's product moment test).

Expression of OPN and its transcriptional regulatory complex including Ets1, Ets2, PEA3, Tcf4 and *β*-catenin were detected by immunohistochemistry and digital scanning microscopy (Figures 1 and 2). Positive correlations between OPN intensity and that of PEA3 (*r*=0.600; *P*<0.01), Ets1 (*r*=0.552; *P*<0.01), Ets2 (*r*=0.521; *P*<0.01) were observed in primary tumours. Osteopontin positivity was generally lower in CRLM in comparison with that of the primary CRC (*P*<0.001; Figure 2, Table 3). The intensity of OPN expression in primary CRCs inversely correlated with the disease-free interval between colectomy and liver resection for metastases (Pearson's correlation coefficient, *r*=−0.123, *P*=0.045; Figure 3). Conversely, expression of each component of the OPN regulatory complex including Ets factors, Tcf4 and *β*-catenin was higher in CRLM than in primary tumours (Table 3). Weakly positive correlations were observed between OPN, Ets1 (*P*=0.047), PEA3 (*P*=0.022), Tcf4 (*P*=0.028) but not Ets2 in CRLM. In the majority of matched pairs, OPN expression was decreased or unchanged in the liver metastasis compared with the primary tumour, whereas Ets1, Ets2, PEA3, Tcf4 and *β*-catenin were predominantly increased (Table 4). The prognostic significance of differential expression of OPN and components of its regulatory complex between primary and secondary tumour was explored by Cox analysis. The ratio of OPN expression between paired samples of primary CRC and CRLM had prognostic significance after liver resection (Table 5).

## Discussion

Osteopontin is an important pro-metastasis gene with complex molecular regulation making its study in human primary and metastatic CRC a particular interest. Ets1, Ets2, PEA3 and Tcf factors bind to specific domains in the OPN promoter (El-Tanani *et al*, 2004, 2010). However, the identification of defined regulatory domains within the OPN promoter is insufficient evidence for involvement of single or combined transcriptional regulators in OPN-driven neoplastic progression. For example, PEA3 is a potent activator of pro-oncogenic OPN (El-Tanani *et al*, 2004) but may also inhibit breast cancer progression (Xing *et al*, 2000). Hence, interaction of coregulators with OPN may be context-specific.

Approximately 20–25% of CRC patients will have detectable liver secondaries at the time of the initial diagnosis and a further 40–50% of patients will typically develop hepatic metastases within 3 years of colectomy (Stangl *et al*, 1994). In this study, patients predominantly had Duke's C and D CRCs at presentation and had a median interval of 18 months between colectomy and liver resection. In this poor prognostic cohort, OPN and its coregulators including Ets factors, *β*-catenin and Tcf4 were detected in primary CRCs and liver metastases by digital microscopy. This method assesses the number of positive pixels as well as the intensity of immunohistochemical staining in annotated areas of histological tissue sections, using specific algorithms (Steinberg and Ali, 2001; Costello *et al*, 2003; Molnar *et al*, 2003). We manually annotated the area of interest corresponding to tumour tissue for digital microscopic assessment. This remains the most accurate method currently available and ensures that marker expression is predominantly confined to tumour cells. Although some background marker expression is inevitable in non-tumour cell fractions of primary and secondary CRC, such quantitative image analysis has superior reproducibility and consistency over observer scoring, in immunohistochemical assays (Ellis *et al*, 2005). By this method, we found that the intensity of OPN expression in primary CRCs, correlated directly with that of key OPN co-activators including PEA3, Ets1 and Ets2 and inversely correlated with the interval until liver resection for CRLM. These findings support prognostic relevance of OPN in CRC, as previously reported (Agrawal *et al*, 2002). Furthermore, our study provides the scientific foundation for further fundamental investigations in model systems, which may accelerate validation of OPN and/or coregulators as molecular targets for therapy.

Signals from an inhospitable tissue microenvironment may influence the growth of metastatic tumour (Fidler, 2002; Nakamura *et al*, 2007). In this study, expression of OPN co-activators including Ets1, Ets2, PEA3 and *β*-catenin were increased in the majority of CRLM *vs* primary CRC. However, these changes were not accompanied by any relative increase in OPN expression, which was unchanged in 21 patients and decreased in 17 CRLMs *vs* primary CRCs. Tcf4 is a potential suppressor of OPN when not complexed with *β*-catenin (El-Tanani *et al*, 2006) and was also increased in CRLM in comparison with primary CRC. However, no significant correlations were observed between Tcf4 and OPN in CRLM. It appears unlikely that Tcf4 alone would have overcome the combinatorial enhancing effects of Ets1, Ets2, PEA3 and *β*-catenin, on OPN expression in CRLM. Although correlations were observed between OPN expression and that of its co-activators in liver metastases, these were weaker than those of primary CRC. Cox analysis demonstrated that the ratio of OPN expression in the primary tumour to that in CRLM (colon to liver ratio) had prognostic significance.

Taken together, our study shows expression differences of OPN and its coregulators between primary CRC and liver metastases. The weaker correlations between OPN and its coregulators in CRLM suggest context specificity of OPN transcriptional control. Unknown signals from the organ microenvironment or additional genetic or epigenetic changes that accumulate in tumour cells as they acquire metastatic capability may also influence OPN regulation, implicated in progression of tumour growth.

## Figures and Tables

**Table 1:** Details of colorectal primary tumours

		**Number**	**%**
Site of colorectal primary	Right colon	12	31.6
	Left colon	13	34.2
	Rectum	13	34.2
Duke's stage	A	0	0.0
	B	5	13.2
	C	13	34.2
	(D)^a^	15	39.5
TNM classification	pT1	0	0.0
(colon specimen)	pT2	0	0.0
	pT3	12	31.6
	pT4	7	18.4
	TNM:T status not formally reported	19	50.0
	N0	7	18.4
	N1	6	15.8
	N2	6	15.8
	TNM:N status not formally reported	19	50.0
	M0	18	47.4
	M1	20	52.6

Abbreviation: TNM=tumour–node–metastases.

a Presented with synchronous metastases.

**Table 2:** Details of resected liver metastases

		**Number**	**%**
Liver resection	Non-anatomical resection	11	28.9
	Left hepatectomy	4	10.5
	Right hepatectomy	20	52.6
	Extended left hepatectomy	1	2.6
	Extended right hepatectomy	2	5.3
Number of liver metastases	1	26	68.4
	2	3	7.9
	3	3	7.9
	4	2	5.3
	>4	4	10.5
Size of largest liver	Median	5	(IQR 4 to 6)
metastasis (cm)	Mode	4	
Liver resection margin	R0	32	84.0
status	R1	6	16.0
Nearest involved	Median	9	(IQR 5 to 10)
margin (mm)	Mode	10	

Abbreviation: IQR=interquartile range.

**Table 3:** Magnitude of differential expression of OPN and coregulators between primary CRC and matched liver metastases

	**Colon (% positivity)**	**Liver (% positivity)**	**Matched pair difference**	**Paired *t*-test significance**
Negative control	2.8±0.3	3.2±0.4	0.5±0.5	0.338
OPN	24.4±2.4	11.7±1.1	−12.7±2.4	<0.001
Ets1	28.6±2.8	59.4±2.2	30.8±3.2	<0.001
Ets2	57.8±2.0	64.2±2.7	6.4±3.4	0.07
PEA3	25.4±2.0	44.2±3.1	18.8±3.3	<0.001
Tcf4	37.3±3.7	48.4±2.7	11.1±4.0	0.009
*β*-catenin	52.7±2.0	62.6±3.1	9.9±3.3	0.006
*β*-Cat/Tcf4 ratio	70.8±6.9	79.7±5.1	−8.9±8.1	0.283

Abbreviations: CRC=colorectal cancer; OPN=osteopontin.

**Table 4:** Direction of change in protein expression between matched colorectal primary and liver metastasis (number of patients)

	**Increased positivity in CRLM**	**Decreased positivity in CRLM**	**Unchanged in CRLM**
OPN	0	17	21
Ets1	28	0	10
Ets2	13	7	18
PEA3	23	4	11
Tcf4	15	5	18
*β*-catenin	18	5	15
*β*-Cat/Tcf4 ratio	8	10	20

Abbreviations: CRLM=colorectal liver metastases; OPN=osteopontin.

**Table 5:** Differential expression of OPN and coregulators between primary and secondary CRC: prognostic significance

			**95% CI for Exp (B)**		
**Marker**	**Mean colon to liver ratio (95% CI)**	**Hazard ratio Exp (B)**	**Lower**	**Upper**	**Significant** ***P-*value**
OPN	3.15 (1.54, 4.75)	1.145	1.002	1.308	0.047
*β*-catenin	0.92 (0.74, 1.10)	5.950	0.471	75.175	0.168
Ets1	0.50 (0.39, 0.62)	0.408	0.031	5.305	0.493
Ets2	1.00 (0.77, 1.22)	0.259	0.030	2.249	0.221
PEA3	0.68 (0.48, 0.87)	0.686	0.136	3.449	0.647
Tcf4	0.85 (0.066, 1.04)	4.520	0.817	24.991	0.084

Abbreviations: CI=confidence interval; CRC=colorectal cancer; CRLM=colorectal liver metastases; OPN=osteopontin.

The prognostic significance of differential expression of OPN and coregulators between primary CRC and liver metastases was assessed by a Cox multivariate proportionate hazard method. Outcomes are expressed as a Hazard ratio (Exp B), which describes effects of a unit increase in colon to liver ratio for each marker on relative risk of death at time *t*, together 95% CIs and significance of the association. The ratio of OPN expression between paired samples of primary CRC and CRLM had prognostic significance after liver resection.
