# Supplementary material for: Expression of osteopontin coregulators in primary colorectal cancer and associated liver metastases
Source: Br J Cancer. 2011 Feb 22;104(6):1007–12. doi: 10.1038/bjc.2011.33 (PMC3065273; doi:10.1038/bjc.2011.33)
Supplement: Supplementary Table S1 [file bjc201133x1.doc]

| **Table S1. ImageScope positive pixel count algorithm inputs** | |
| --- | --- |
| **View Width** | 1000 |
| **View Height** | 1000 |
| **Overlap Size** | 0 |
| **Image Zoom** | 1.0 |
| **Pixel Area (millimeter-squared)** | 2.50E-07 |
| **Hue Value (Center)** | .1 |
| **Hue Width** | .5 |
| **Color Saturation Threshold** | 4.00E-02 |
| **Intensity Threshold WEAK (Upper Limit)** | 220 |
| **Intensity Threshold WEAK (Lower Limit)** | 175 |
| **Intensity Threshold MEDIUM (Upper Limit)** | 175 |
| **Intensity Threshold MEDIUM (Lower Limit)** | 100 |
| **Intensity Threshold STRONG (Upper Limit)** | 100 |
| **Intensity Threshold STRONG (Lower Limit)** | 0 |
| **Intensity Threshold Negative Pixels** | -1 |
|  |  |
